# Gene Expression in Experimental Aortic Coarctation and Repair: Candidate Genes for Therapeutic Intervention?

**DOI:** 10.1371/journal.pone.0133356

**Published:** 2015-07-24

**Authors:** John F. LaDisa, Serdar Bozdag, Jessica Olson, Ramani Ramchandran, Judy R. Kersten, Thomas J. Eddinger

**Affiliations:** 1 Department of Biomedical Engineering, Marquette University, Milwaukee, Wisconsin, United States of America; 2 Department of Medicine, Division of Cardiovascular Medicine, Medical College of Wisconsin, Milwaukee, Wisconsin, United States of America; 3 Biotechnology and Bioengineering Center, Medical College of Wisconsin, Milwaukee, Wisconsin, United States of America; 4 Herma Heart Center, Children’s Hospital of Wisconsin, Milwaukee, Wisconsin, United States of America; 5 Department of Mathematics, Statistics, and Computer Science, Marquette University, Milwaukee, Wisconsin, United States of America; 6 Department of Physiology, Medical College of Wisconsin, Milwaukee, Wisconsin, United States of America; 7 Departments of Pediatrics and Obstetrics and Gynecology, Medical College of Wisconsin and the Developmental Vascular Biology Program, Children’s Hospital of Wisconsin, Milwaukee, Wisconsin, United States of America; 8 Department of Anesthesiology, Medical College of Wisconsin, Milwaukee, Wisconsin, United States of America; 9 Department of Pharmacology and Toxicology, Medical College of Wisconsin, Milwaukee, Wisconsin, United States of America; 10 Department of Biological Sciences, Marquette University, Milwaukee, Wisconsin, United States of America; University of Jaén, SPAIN

## Abstract

Coarctation of the aorta (CoA) is a constriction of the proximal descending thoracic aorta and is one of the most common congenital cardiovascular defects. Treatments for CoA improve life expectancy, but morbidity persists, particularly due to the development of chronic hypertension (HTN). Identifying the mechanisms of morbidity is difficult in humans due to confounding variables such as age at repair, follow-up duration, coarctation severity and concurrent anomalies. We previously developed an experimental model that replicates aortic pathology in humans with CoA without these confounding variables, and mimics correction at various times using dissolvable suture. Here we present the most comprehensive description of differentially expressed genes (DEGs) to date from the pathology of CoA, which were obtained using this model. Aortic samples (n=4/group) from the ascending aorta that experiences elevated blood pressure (BP) from induction of CoA, and restoration of normal BP after its correction, were analyzed by gene expression microarray, and enriched genes were converted to human orthologues. 51 DEGs with >6 fold-change (FC) were used to determine enriched Gene Ontology terms, altered pathways, and association with National Library of Medicine Medical Subject Headers (MeSH) IDs for HTN, cardiovascular disease (CVD) and CoA. The results generated 18 pathways, 4 of which (cell cycle, immune system, hemostasis and metabolism) were shared with MeSH ID’s for HTN and CVD, and individual genes were associated with the CoA MeSH ID. A thorough literature search further uncovered association with contractile, cytoskeletal and regulatory proteins related to excitation-contraction coupling and metabolism that may explain the structural and functional changes observed in our experimental model, and ultimately help to unravel the mechanisms responsible for persistent morbidity after treatment for CoA.

## Introduction

Coarctation of the aorta (CoA) is a congenital defect during which the proximal descending thoracic aorta (dAo) is significantly narrowed, and is one of the most common congenital heart defects in the U.S (5,000 to 8,000 births annually)[1, 2]. Catheter-based treatments are available, but surgery is the treatment of choice in infancy due to its excellent short-term outcomes[3, 4]. The focal narrowing of coarctation has led some researchers to believe CoA is a *simple* disease[5] that can be alleviated by correction of the associated blood pressure (BP) gradient. However, the natural history of CoA suggests otherwise, as patients often have a reduced life expectancy from increased cardiovascular morbidity. The most notable complication of CoA is hypertension (HTN)[3], but other common sources of morbidity include early onset coronary artery disease and the potential for cerebral and/or aortic aneurysms. For example, even after successful treatment ~1/3 of CoA patients become hypertensive in adolescence[6], and the prevalence of HTN increases to 90% by 50–70 years of age[7], often despite pharmacological therapy.

Identifying the causes of increased morbidity in humans with corrected CoA is difficult for several reasons. Causal genetic contributors to the formation of CoA are extremely difficult to isolate from changes in gene expression due to the mechanical stimuli that are introduced by the coarctation itself once the ductus arteriosus closes shortly after birth. The ability to separate these two potential contributors to long term cardiovascular (CV) morbidity in CoA would provide added clarity when interpreting experimental results, and two potentially distinctive routes for clinical translation. Moreover, the relatively small number of CoA patients treated at a given center each year makes it difficult to design studies that will control for their heterogeneity from confounding variables such as: differences in age at repair; time to follow-up; severity of coarctation before repair; and concomitant CV anomalies. To address these challenges, we developed a novel animal model of CoA that allows for control of these variables, eliminates genetic predispositions at the onset of the disease, and introduces mechanical stimuli caused by CoA using a clinically-representative 20 mmHg BP gradient[8]. This model is devoid of concomitant anomalies such as bicuspid aortic valve, transverse arch hypoplasia, and septal defects. The model also mimics thoracic aortic changes presenting in humans with CoA[9–11], and uniquely allows for the study of corrected CoA through the use of dissolvable sutures to induce the coarctation.

A summary of previous findings using this model[12] are provided in Table 1. While the stimuli for vascular alterations and coarctation-induced morbidity are reversed for the equivalent of 6 human years after the suture dissolves in corrected rabbits, data from this model shows restoration of normal BP alone does not alleviate increases in medial thickness and stiffness, or decreases in contractility to phenylephrine (PE) and endothelial cell (EC) dysfunction as measured by acetylcholine (ACh) vs sodium nitroprusside induced relaxation. Immunohistochemical results show a shift from smooth muscle (SM) to non-muscle (NM) myosin heavy chain (MHC) isoform expression in the medial smooth muscle cells (SMCs) indicating a change in phenotype from contractile to synthetic for both CoA and corrected rabbits[12]. These changes are consistent with findings in humans[9–11]. For example, evidence for impaired function includes an increased response to norepinephrine (NE) in the brachial, but not femoral, artery of CoA vs. control patients[13]; abnormal brachial but not femoral function in repaired CoA patients[14]; and increased collagen and stiffness with less SM but greater contractility to potassium and NE stimulation in upstream vessels of CoA patients[15]. Importantly, none of the changes in vascular structure or function return to control levels after correction of the CoA in our model, but associated mechanisms have not yet been fully elucidated. The objective of the current investigation was to use microarray techniques to quantify differentially expressed genes (DEGs) in the upstream aorta subjected to high arterial BP after surgical induction of CoA, and restoration of normal arterial BP after its correction. DEGs may offer additional insight into potential mechanisms of persistent CV morbidity despite successful surgical repair.

**Table 1:** Summary of structural and functional changes to date from a novel rabbit model of native and corrected CoA (from Menon et al—Am J Physiol Heart Circ Physiol. 2012; 303:H1304-18).

Changes vs. control		Proximal to CoA		Distal to CoA	
		CoA	Corrected	CoA	Corrected
*Altered Hemodynamics*	BP	↑	NC	NC	NC
	Wall shear stress	↓	NC	↑↑	↓
	Strain	↓↓	↓	N/A	N/A
*Vascular Remodeling*	Medial thickness	↑↑	↑	NC	NC
	Altered SM Phenotype	↑	↑	NC	NC
*Endothelial Dysfunction*	Endothelial- derived relaxation	↓↓	↓	↓	NC
	SM-derived relaxation	NC	NC	↑	NC
*Decreased Contractility*	Vessel contractility	↓	↓	NC	↓
	Vascular stiffness	↑↑	↑	N/A	N/A

NC = no change relative to control, N/A = not available; SM = smooth muscle; multiple arrows indicates a more pronounced change.

## Materials and Methods

### Ethics statement

This study was carried out in accordance with the recommendations in the Guide for the Care and Use of Laboratory Animals of the National Institutes of Health. The protocol was approved by the Committee on the Ethics of Animal Experiments of the Medical College of Wisconsin (protocol AUA1175) and Marquette University (protocol AR0223). Euthanasia was administered by intravenous overdose of pentobarbital sodium (100 mg/kg) consistent with the American Veterinary Medical Association guidelines for the euthanasia of animals.

### Experimental protocol, tissue harvest and sample preparation

**Fig 1:**
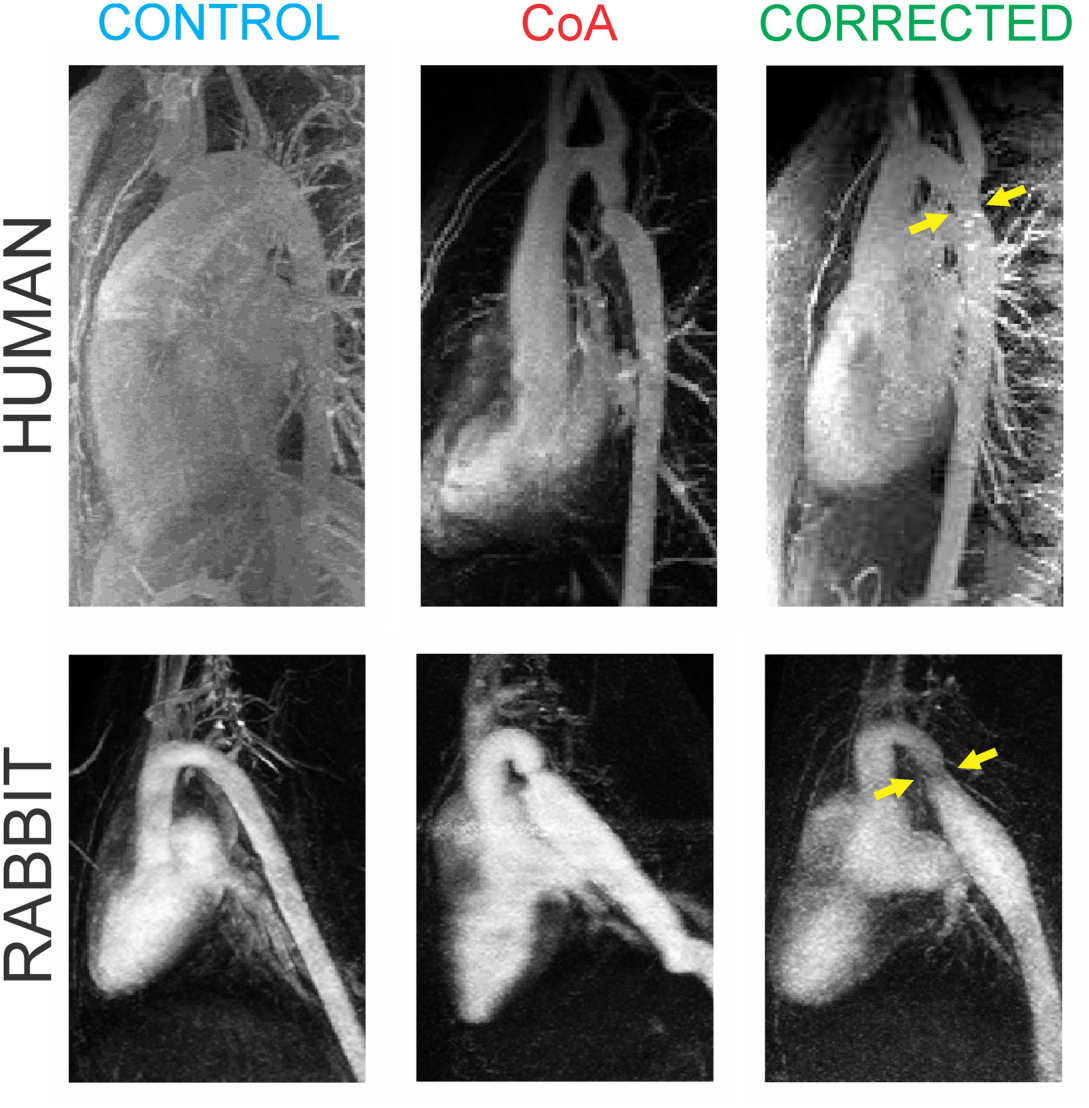
Images of morphological similarity between untreated and corrected CoA in humans (top row) and our rabbit model (bottom row). Arrows show correction sites. Human images are adapted from related studies discussed in detail in LaDisa et al.—Congenit Heart Dis. 2011 Sep; 6(5): 432–43 and LaDisa and Figueroa et al—J. Biomech. Eng. 2011 Sep;133(9):091008.

Since the incidence of CoA is greater in males (2:1), male New Zealand white rabbits ~10 weeks old and weighing ~1.0 kg were randomly designated to undergo proximal dAo CoA. Studies dating back over 40 years suggested the majority of morbidity for CoA can be explained on the basis of abnormal hemodynamics through the ascending aorta and its branches (i.e. proximal to the coarcation) by showing that its conduit (blood flow) and cushioning (capacitance) functions were altered by CoA[16]. This is consistent with HTN often still observed during rest and exercise in patients with CoA. Other studies[17, 18] did not find convincing evidence for elevated plasma renin activity or angiotensin II levels[19] in CoA patients. These findings suggest increased plasma renin activity is not the primary cause of elevated BP leading to vascular dysfunction in CoA, and have helped set our focus on the proximal aorta and its branches. A 20 mmHg BP gradient was imposed for the current investigation using silk (permanent) or Vicryl (degradable) suture as previously described[20] to mimic untreated CoA and surgically corrected CoA, respectively. A 20 mmHg BP gradient is the putative value for when treatment of CoA is warranted[8]. Moreover, BP is very similar across species[21], making the stimuli for remodeling from our rabbit model analogous to that in human CoA. Similar to the human condition of CoA with the closure of the ductus arteriosus 1–7 days after birth, rabbits develop a pronounced stenosis and accompanying elevated BP as a stimulus for arterial remodeling within one week. Rabbits undergoing coarctation with degradable suture develop an initial stenosis similar to CoA rabbits. However, degradation of Vicryl suture restores aortic diameter close to normal, but with modest residual narrowing mimicking morphology often observed after surgical resection with end-to-end anastomosis in humans (Fig 1). According to the manufacturer, this suture is completely absorbed after ~9 weeks with an initial strength of approximately 15 lbf and a known strength retention curve based on the number of days since suture use. An analysis using this retention curve, with knowledge of rabbit aortic diameters, indicates that the force exerted on the suture used to create the coarctation by integrating the distribution of tractions within this region[22] exceeds the strength indicated by the manufacturer after ~21 days. Importantly, this information suggests that our novel approach of inducing CoA with dissolvable Vicryl suture provides the stimulus of altered hemodynamics from CoA for three weeks before restoring BP to normal for >4 months (i.e., ~6 human years) before the experimental end point of 32 weeks of age used in this study. Non-experimental rabbits were also designated for a control group. A sham group was not included to limit the number of animals used to only those that are truly necessary, consistent with IACUC recommendations (three “Rs”—reduce, refine, replace). Our prior work with this model of CoA and correction[12] revealed a statistically significant increase in mean, systolic and pulse BP proximal to the coarctation for CoA as compared to both control and corrected rabbits. A ~4 mm circumferential region from the proximal aorta between the coarctation site and left subclavian artery was subsequently excised at harvest and frozen. These frozen samples (n = 4/group) were shipped overnight to Arraystar, Inc (Rockville, MD) for microarray analysis.

### Microarray analysis

The Agilent Array platform was employed for analysis using a Whole Genome Oligo transcriptome-wide rabbit (Oryctolagus cuniculus) microarray (model G2519F-020908) to study gene expression profiling at the global level, and enhance the understanding of biology and gene functions. 43,803 rabbit probes are represented with content sourced from RefSeq (Release 29; May 2008), UniGene (Build 11; Mar 2008) and Ensembl (Release 49; Feb 2008). The microarray slide was printed using Agilent's 60-mer SurePrint technology and used according to manufacturer literature. Briefly, sample labeling and hybridization were performed using the Agilent One-Color Microarray-Based Gene Expression Analysis protocol. Total RNA from each sample was linearly amplified and labeled with Cy3-UTP. The labeled cRNAs were purified by RNeasy Mini Kit (Qiagen). The concentration and specific activity of the labeled cRNAs (pmol Cy3/μg cRNA) were measured by NanoDrop ND-1000. 1 μg of each labeled cRNA was fragmented by adding 11 μl 10 × blocking agent and 2.2 μl of 25 × fragmentation buffer, then heated at 60°C for 30 min, and 55 μl 2 × GE buffer was added to dilute the labeled cRNA. 100μl of hybridization solution was dispensed into the gasket slide and assembled to the gene expression microarray slide. The slides were incubated for 17 hours at 65°C in an Agilent Hybridization Oven. The hybridized arrays were washed, fixed and scanned with using the Agilent DNA Microarray Scanner (model G2505C).

Agilent Feature Extraction software (version 11.0.1.1) was used to analyze acquired array images. Quantile normalization and subsequent data processing were performed using the GeneSpring GX v11.5.1 software (Agilent Technologies). Probes for which at least 8 out of 12 samples had flags in Detected (“All Targets Value”) were then chosen for further data analysis. Differentially expressed probes with statistical significance were identified through Volcano Plot filtering. Finally, hierarchical clustering was performed to show the distinguishable gene expression profiling among samples. The data discussed in this publication have been deposited in NCBI's Gene Expression Omnibus[23] and are accessible through GEO Series accession number GSE70687 (http://www.ncbi.nlm.nih.gov/geo/query/acc.cgi?acc=GSE70687).

### Analysis of DEGs

**Fig 2:**
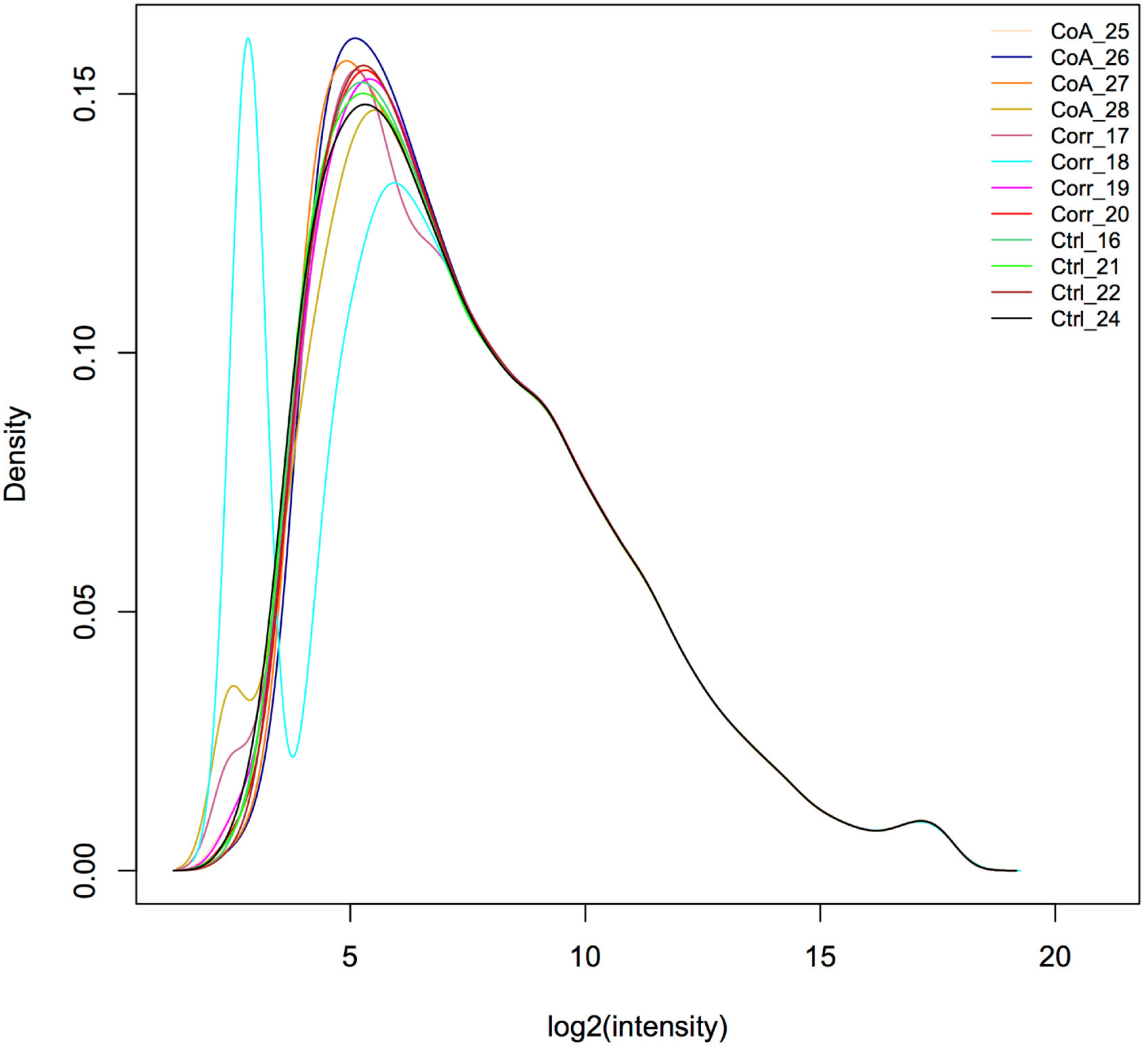
Normalized intensity distributions from kernel density plots indicate the sample with a bimodal distribution (Corrected rabbit #18) is an outlier. This sample was therefore excluded from the analysis indicated by the remaining figures and tables.

Normalized intensity distributions from kernel density plots indicated that the sample from one rabbit was an outlier (Fig 2). This sample was therefore excluded from the analysis. Comparison of gene probes was made between groups of samples in three ways: (1) CoA vs Control, (2) Corrected vs Control, and (3) CoA vs Corrected. Probes within each comparison were then ordered by their expression levels for both up and down-regulation, and those with a >6 fold change (FC) in gene expression and a *p*-value <0.05 were compared using Venny[24] similar to several previously published reports[25, 26]. Highly expressed probes for genes common to CoA vs Control and Corrected vs Control are particularly interesting as they could help explain sources of morbidity persisting after restoring BP, as could probes for highly expressed genes in CoA vs Corrected that are not present in the CoA vs Control comparison. Unfortunately, the rabbit genome is poorly annotated thereby limiting rapid functional analysis and straightforward investigation of pathways associated with a set of gene probes. We therefore used Better Bunny (http://cptweb.cpt.wayne.edu)[27], an online functional annotation tool using data derived from public bioinformatics resources including NCBI and Ensembl databases, to map probes for DEGs to highly homologous human genes. Agilent rabbit microarray probe identifiers from common interesting regions of the comparisons mentioned above were input to Better Bunny, and corresponding human orthologue genes were output in Ensembl Gene (ensembl gene stable ID) format using an identity threshold >50%[28].

Ensemble Gene IDs from Better Bunny were used in several ways. First, they were input into the WEB-based GEne SeT AnaLysis Toolkit (WebGestalt) to perform functional analysis[29]. Specifically, DEGs with >6 FC in the regions of interest mentioned were characterized using the Gene Ontology (GO) analysis function in WebGestalt in order to elucidate important GO terms in cellular component, molecular function and biological process domains. All analyses conducted in WebGestalt used the following parameters: organism = hsapiens; reference set = entrezgene; significance level = 0.05; statistical method = Hypergeometric; multiple test adjustment = Benjamini-Hochberg; minimum number of genes for a category = 2. Second, DEGs with >6 FC in the regions of interest were compared to a list of candidate genes for hypertension in the Text-mined Hypertension, Obesity and Diabetes (T-HOD) database[30] and Comparative Toxicogenomics Database (http://ctdbase.org/). Third, pathways containing ≥5 DEGs (each with >6 FC), which were also associated with National Library of Medicine Medical Subject Headers (MeSH) disease IDs of hypertension (MESH:D006973), cardiovascular disease (CVD; MESH:D002318) or CoA (MESH:D001017), were found using the Integrated Pathway Analysis Database for Systematic Enrichment Analysis (IPAD)[31] and Ingenuity Pathway Analysis (IPA, Redwood City, CA). Lastly, a thorough literature search was conducted in PubMed for all DEGs with >6 FC to review the available literature and further appreciate their potential involvement in sources of CV morbidity in CoA.

### Messenger RNA analysis by quantitative reverse transcriptase PCR (qRT-PCR)

In order to validate microarray results, total RNA samples were analyzed using qRT-PCR. cDNA was synthesized using the RT^2^ First Strand kit (Qiagen) according to the manufacturer’s instructions. Genomic DNA was eliminated by a brief incubation of total RNA with GE Buffer. Samples were chilled and mixed with 5X RT Buffer 3, Primer and External Control Mix, RT Enzyme Mix 3, and RNase-free water. The mixture was then incubated at 42°C for fifteen minutes, then at 95°C for five minutes. qRT-PCR was conducted using the iCycler Real-Time PCR detection system (BioRad).

Expression of messenger RNAs were analyzed in technical triplicate using RT^2^ Primer Assays (SABiosciences) for DEGs with the most pronounced expression (e.g. ITGA4 and UCP1). cDNA was diluted and combined with RT^2^ SYBR Green/Fluorescein Master Mix. Samples were exposed to an initial 95°C hot-start activation step, followed by 40 cycles of 94°C denaturation and 60° annealing/extension phases. Data collected from these experiments defined Ct values of the mRNAs present in each sample. Expression levels of the housekeeping gene GAPDH were used as controls to normalize samples. Samples without reverse transcriptase and melt curve denaturation were also run to confirm sample purity.

## Results

**Fig 3:**
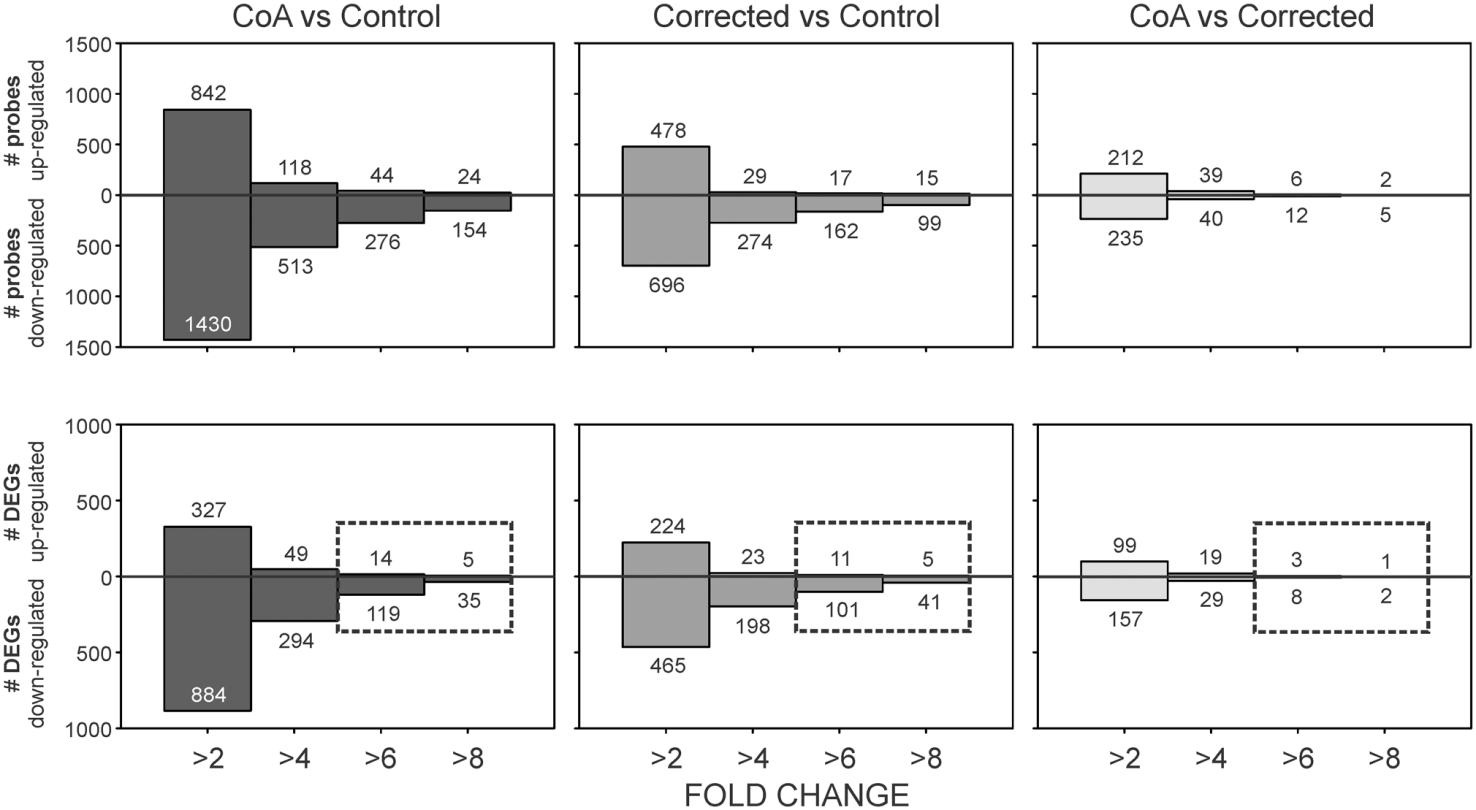
(Top) Probes with >2 fold-change and a p-value <0.05 for each comparison were binned based on their expression levels using a minimum of 2, 4, 6 and >8 fold changes. Human genes corresponding to expressed probes on the rabbit chip were determined using orthologue analysis in Better Bunny (Craig et al.—BMC Bioinformatics. 2012 May 8;13:84). Probes without an orthologue human gene and redundant differentially expressed genes (DEGs) were omitted. (Bottom) Unique human orthologue DEGs with >6 FC (indicated within the boxes below) were further studied through GO term, function and pathway analysis, and extensive literature review.

Differentially expressed probes for each comparison (i.e. CoA vs Control, Corrected vs Control, and CoA vs Corrected) received from Arraystar were binned based on their expression levels using a minimum of 2, 4, 6 and >8 fold changes (Fig 3). A total of 2,272 and 1,174 probes corresponding to genes with >2 FC were found for the CoA vs Control and Corrected vs Control comparisons, respectively. The CoA vs Corrected comparison yielded substantially fewer probes (447) with >2 FC. Human orthologue analysis of probes with >2 FC in Better Bunny revealed 1,211, 689 and 256 unique DEGs in the CoA vs Control, Corrected vs Control, and CoA vs Corrected comparisons, respectively. When scrutinized at a >6 FC level, the number of DEGs reduced to 133, 112 and 11 for the CoA vs Control, Corrected vs Control, and CoA vs Corrected comparisons, respectively (Fig 3).

**Fig 4:**
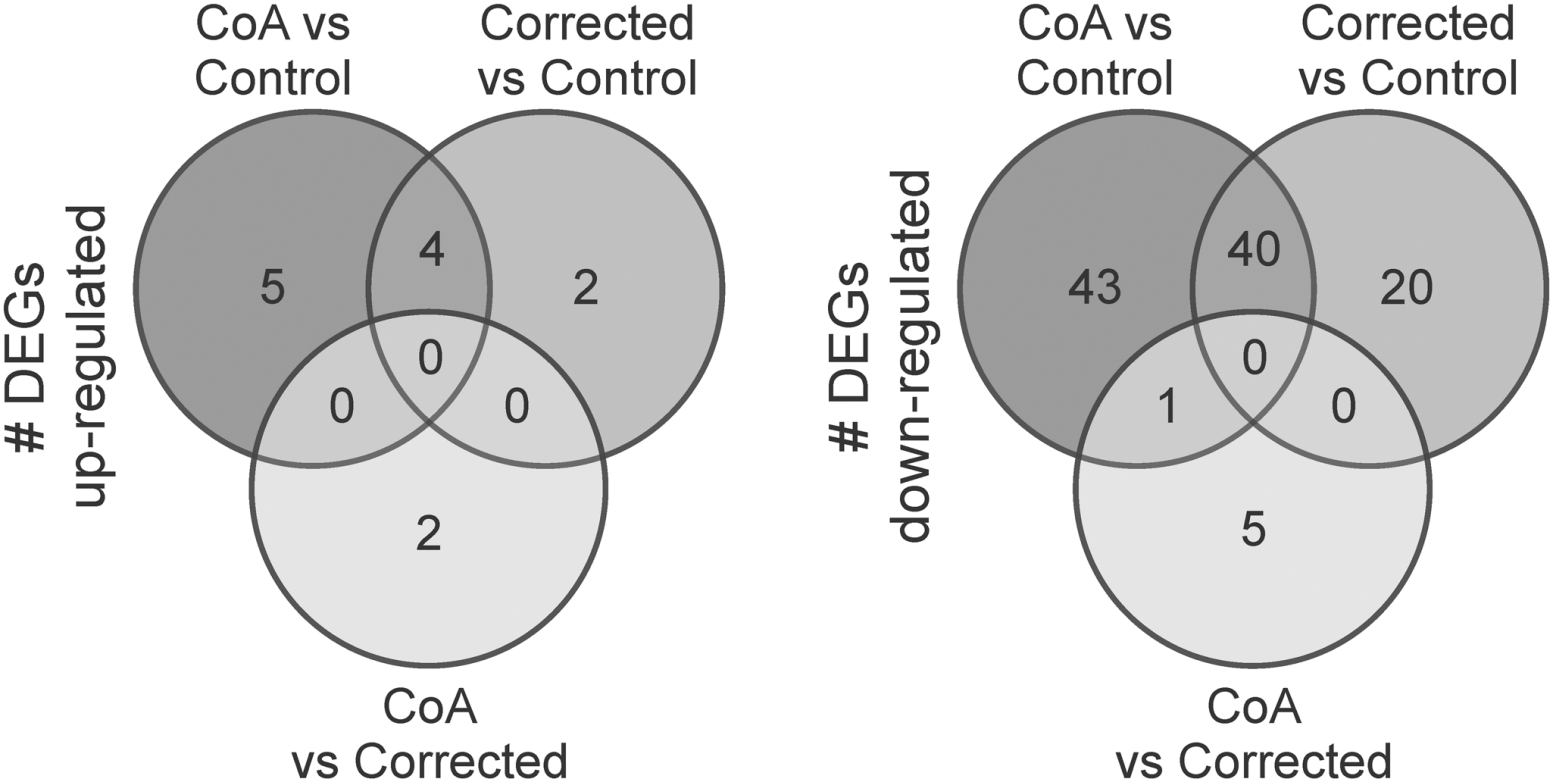
Venn Diagrams of DEGs with >6 FC. Probes common to the CoA vs Control and Corrected vs Control comparisons could help explain persistent morbidity after restoring BP, as could highly expressed probes in the CoA vs Corrected comparison that are not found in CoA vs Control comparison.

**Fig 5:**
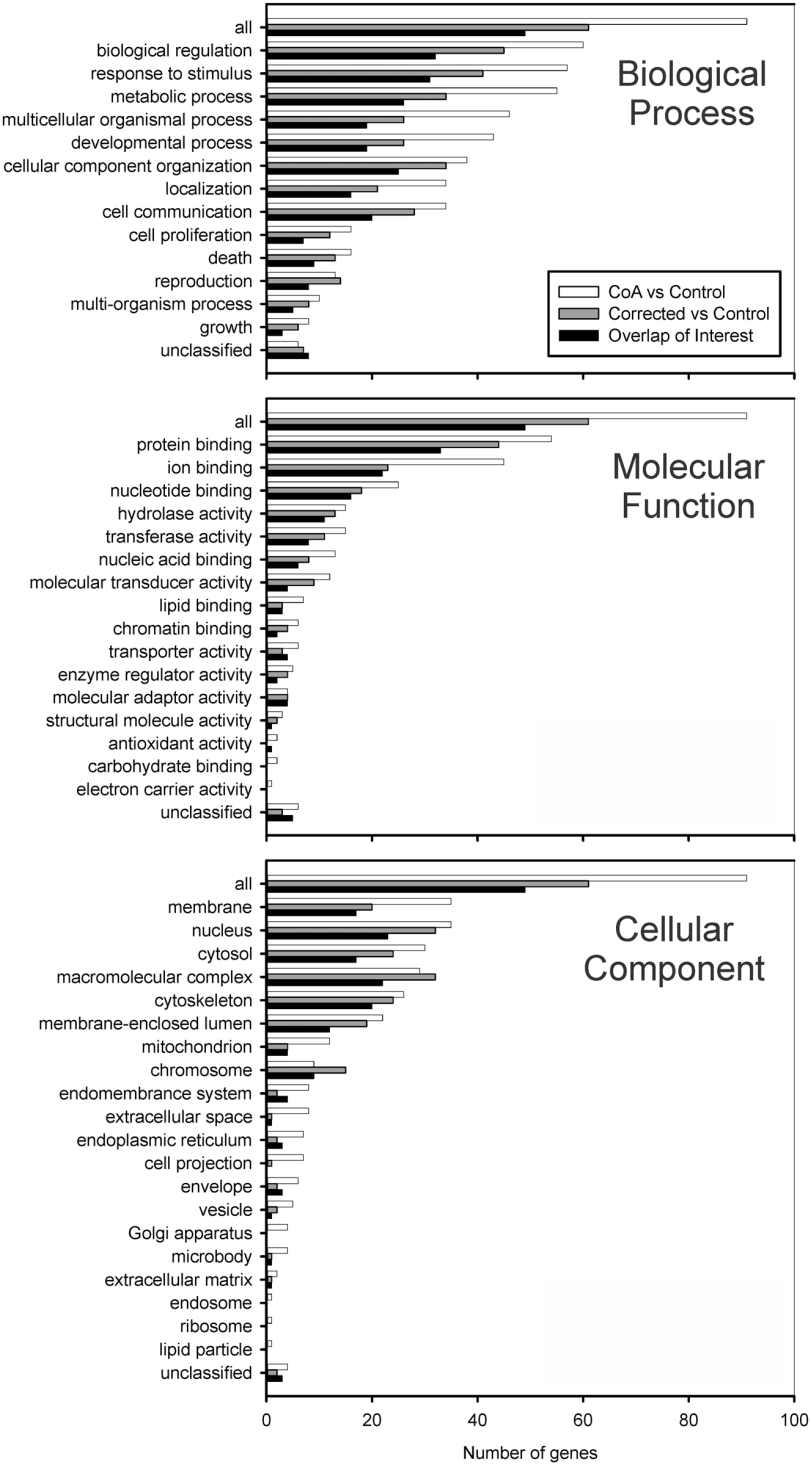
Gene Ontology (GO) terms for DEGs with >6 FC are shown by biological process (top), molecular function (middle), and cellular component (bottom) domains. Comparison of DEGs was made between groups of samples in three ways: (1) CoA vs Control, (2) Corrected vs Control, and (3) CoA vs Corrected. DEGs common to CoA vs Control and Corrected vs Control are particularly interesting as they could help explain sources of morbidity persisting after restoring BP, as could probes for highly expressed genes in CoA vs Corrected that are not present in the CoA vs Control comparison. These DEGs of potential interesting regions are indicated by black bars, referring back to overlapping DEGs of interest in Fig 4.

Venn diagrams of up and down-regulated DEGs with >6 FC common to the CoA vs Control and Corrected vs Control comparisons, as well as DEGs with >6 FC in the CoA vs Corrected comparison that were not found in CoA vs Control comparison, are shown in Fig 4. Details for these DEGs are provided in Table 2 and may be of particular interest in the etiology of morbidity persisting after correction of CoA. GO terms for DEGs with >6 FC within these regions of interest are shown by biological process, molecular function, and cellular component domains in Fig 5.

**Table 2:** DEGs that may be of particular interest (referring to Fig 4) in the morbidity persisting after correction of CoA.

**Upregulated**				
***Common elements in "CoA v Cont" and "Corr v Cont"*:**				
*Human Ensembl Gene ID*	*Gene Name*	*Human Description*	**>6FC**	**>8FC**
ENSG00000241644	INMT	indolethylamine N-methyltransferase [Source:HGNC Symbol;Acc:6069]	x	
ENSG00000172399	MYOZ2	myozenin 2 [Source:HGNC Symbol;Acc:1330]	x	x
ENSG00000133110	POSTN	periostin, osteoblast specific factor [Source:HGNC Symbol;Acc:16953]	x	x
ENSG00000118194	TNNT2	troponin T type 2 (cardiac) [Source:HGNC Symbol;Acc:11949]	x	x
		**count for each FC**	**4**	**3**
***Elements only in "CoA vs Corr"*:**				
ENSG00000121053	EPX	eosinophil peroxidase [Source:HGNC Symbol;Acc:3423]	x	x
ENSG00000091490	SEL1L3	sel-1 suppressor of lin-12-like 3 (C. elegans) [Source:HGNC Symbol;Acc:29108]	x	
		**count for each FC**	**2**	**1**
**Downregulated**				
***Common elements in "CoA v Cont" and "Corr v Cont"*:**				
ENSG00000158874	APOA2	apolipoprotein A-II [Source:HGNC Symbol;Acc:601]	x	x
ENSG00000066279	ASPM	asp (abnormal spindle) homolog, microcephaly associated (Drosophila) [Source:HGNC Symbol;Acc:19048]	x	
ENSG00000178999	AURKB	aurora kinase B [Source:HGNC Symbol;Acc:11390]	x	x
ENSG00000156970	BUB1B	budding uninhibited by benzimidazoles 1 homolog beta (yeast) [Source:HGNC Symbol;Acc:1149]	x	
ENSG00000157456	CCNB2	cyclin B2 [Source:HGNC Symbol;Acc:1580]	x	
ENSG00000198821	CD247	CD247 molecule [Source:HGNC Symbol;Acc:1677]	x	
ENSG00000160654	CD3G	CD3g molecule, gamma (CD3-TCR complex) [Source:HGNC Symbol;Acc:1675]	x	x
ENSG00000117399	CDC20	cell division cycle 20 homolog (S. cerevisiae) [Source:HGNC Symbol;Acc:1723]	x	
ENSG00000184661	CDCA2	cell division cycle associated 2 [Source:HGNC Symbol;Acc:14623]	x	
ENSG00000170312	CDK1	cyclin-dependent kinase 1 [Source:HGNC Symbol;Acc:1722]	x	
ENSG00000035499	DEPDC1B	DEP domain containing 1B [Source:HGNC Symbol;Acc:24902]	x	x
ENSG00000174371	EXO1	exonuclease 1 [Source:HGNC Symbol;Acc:3511]	x	
ENSG00000188820	FAM26F	family with sequence similarity 26, member F [Source:HGNC Symbol;Acc:33391]	x	x
ENSG00000162654	GBP4	guanylate binding protein 4 [Source:HGNC Symbol;Acc:20480]	x	
ENSG00000177602	GSG2	germ cell associated 2 (haspin) [Source:HGNC Symbol;Acc:19682]	x	
ENSG00000161405	IKZF3	IKAROS family zinc finger 3 (Aiolos) [Source:HGNC Symbol;Acc:13178]	x	
ENSG00000115232	ITGA4	integrin, alpha 4 (antigen CD49D, alpha 4 subunit of VLA-4 receptor) [Source:HGNC Symbol;Acc:6140]	x	
ENSG00000166803	KIAA0101	KIAA0101 [Source:HGNC Symbol;Acc:28961]	x	
ENSG00000138160	KIF11	kinesin family member 11 [Source:HGNC Symbol;Acc:6388]	x	x
ENSG00000137807	KIF23	kinesin family member 23 [Source:HGNC Symbol;Acc:6392]	x	
ENSG00000090889	KIF4A	kinesin family member 4A [Source:HGNC Symbol;Acc:13339]	x	
ENSG00000182866	LCK	lymphocyte-specific protein tyrosine kinase [Source:HGNC Symbol;Acc:6524]	x	x
ENSG00000165304	MELK	maternal embryonic leucine zipper kinase [Source:HGNC Symbol;Acc:16870]	x	
ENSG00000121152	NCAPH	non-SMC condensin I complex, subunit H [Source:HGNC Symbol;Acc:1112]	x	
ENSG00000143228	NUF2	NUF2, NDC80 kinetochore complex component, homolog (S. cerevisiae) [Source:HGNC Symbol;Acc:14621]	x	x
ENSG00000078589	P2RY10	purinergic receptor P2Y, G-protein coupled, 10 [Source:HGNC Symbol;Acc:19906]	x	
ENSG00000183918	SH2D1A	SH2 domain containing 1A [Source:HGNC Symbol;Acc:10820]	x	x
ENSG00000154839	SKA1	spindle and kinetochore associated complex subunit 1 [Source:HGNC Symbol;Acc:28109]	x	
ENSG00000141293	SKAP1	src kinase associated phosphoprotein 1 [Source:HGNC Symbol;Acc:15605]	x	
ENSG00000140284	SLC27A2	solute carrier family 27 (fatty acid transporter), member 2 [Source:HGNC Symbol;Acc:10996]	x	
ENSG00000076382	SPAG5	sperm associated antigen 5 [Source:HGNC Symbol;Acc:13452]	x	
ENSG00000117632	STMN1	stathmin 1 [Source:HGNC Symbol;Acc:6510]	x	
ENSG00000121895	TMEM156	transmembrane protein 156 [Source:HGNC Symbol;Acc:26260]	x	
ENSG00000131747	TOP2A	topoisomerase (DNA) II alpha 170kDa [Source:HGNC Symbol;Acc:11989]	x	
ENSG00000229164	TRAC	T cell receptor alpha constant [Source:HGNC Symbol;Acc:12029]	x	
ENSG00000163519	TRAT1	T cell receptor associated transmembrane adaptor 1 [Source:HGNC Symbol;Acc:30698]	x	
ENSG00000211772	TRBC2	T cell receptor beta constant 2 [Source:HGNC Symbol;Acc:12157]	x	
ENSG00000112742	TTK	TTK protein kinase [Source:HGNC Symbol;Acc:12401]	x	
ENSG00000175063	UBE2C	ubiquitin-conjugating enzyme E2C [Source:HGNC Symbol;Acc:15937]	x	x
ENSG00000109424	UCP1	uncoupling protein 1 (mitochondrial, proton carrier) [Source:HGNC Symbol;Acc:12517]	x	x
		**count for each FC**	**40**	**11**
***Elements only in "CoA vs Corr"*:**				
ENSG00000116748	AMPD1	adenosine monophosphate deaminase 1 [Source:HGNC Symbol;Acc:468]	x	
ENSG00000196296	ATP2A1	ATPase, Ca++ transporting, cardiac muscle, fast twitch 1 [Source:HGNC Symbol;Acc:811]	x	x
ENSG00000130813	C19orf66	chromosome 19 open reading frame 66 [Source:HGNC Symbol;Acc:25649]	x	
ENSG00000109061	MYH1	myosin, heavy chain 1, skeletal muscle, adult [Source:HGNC Symbol;Acc:7567]	x	x
ENSG00000184271	POU6F1	POU class 6 homeobox 1 [Source:HGNC Symbol;Acc:9224]	x	
		**count for each FC**	**5**	**2**
		***Total genes for consideration based on FC selected***	***51***	***17***

**Fig 6:**
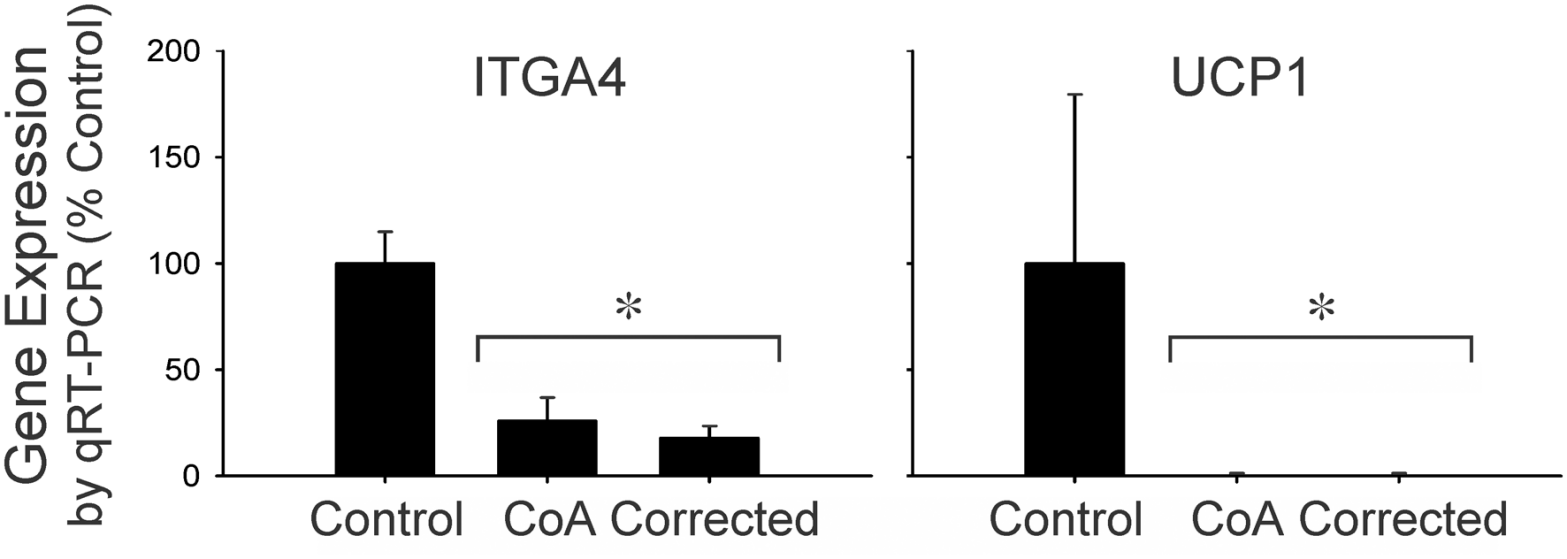
Verification of expression by qRT-PCR for two of the genes with the most pronounced decrease in expression by microarray analysis, ITGA4 and UCP1. * = significantly different (P<0.05) from Control.

There were six genes in common between DEGs revealed by human orthologue analysis and the T-HOD database (Table 3). Of these genes, uncoupling protein 1 (UCP1) was down-regulated 21.4–21.5 fold for CoA vs Control comparison, and down-regulated 11.9–14.9 fold for the Corrected vs Control comparison. This pronounced decrease in expression vs control, as well as that of ITGA4, was also verified by qRT-PCR (Fig 6). For DEGs with >6 FC, integrin, alpha 4—antigen CD49D, alpha 4 subunit of VLA-4 receptor (ITGA4) and sperm associated antigen 5 (SPAG5) were found in the down-regulated region of overlap between the CoA vs Control and Corrected vs Control. When lowering the FC cutoff to >4, three additional DEGs in common with hypertension gene candidates from the T-HOD database appeared. Those in the down-regulated region of overlap between the CoA vs Control and Corrected vs Control overlap included chemokine receptor 4 (CXCR4) and v-src sarcoma viral oncogene homolog (SRC). Regulator of calcineurin 1 (RCAN1) also presented, but in the non-overlapping region of CoA vs Corrected. Nineteen of the DEGs (>2 FC) from the current investigation were associated with aortic coarctation or its descendants in the Comparative Toxicogenomics Database. Thirteen of these DEGs were also associated with MeSH IDs for CoA or hypertension (Table 4).

**Table 3:** Genes in common between DEGs revealed by human orthologue analysis of rabbit microarray probes with >4, >6 or >8 fold change, and the hypertension portion of the T-HOD database.

Gene	Name	FC	regulated	Overlapping Region
UCP1	uncoupling protein 1 (mitochondrial, proton carrier)	>8	down	CoA vs Control & Corrected vs Control
ITGA4	integrin, alpha 4—antigen CD49D, alpha 4 subunit of VLA-4 receptor	>6	down	CoA vs Control & Corrected vs Control
SPAG5	sperm associated antigen 5	>6	down	CoA vs Control & Corrected vs Control
CXCR4	chemokine (C-X-C motif) receptor 4	>4	down	CoA vs Control & Corrected vs Control
SRC	v-src sarcoma (Schmidt-Ruppin A-2) viral oncogene homolog	>4	down	CoA vs Control & Corrected vs Control
RCAN1	regulator of calcineurin 1	>4	down	CoA v Control only

**Table 4:** Genes in common between DEGs of interest (i.e. from Venn diagrams) revealed by human orthologue analysis of rabbit microarray probes with >2 fold change, and genes associated with aortic coarctation or its descendants from the Comparative Toxicogenomics Database. Bolded DEGs are also associated with disease MESHIDs for CoA (for DEGs >2FC) or HTN (for DEGs >6FC) according to the Integrated Pathway Analysis Database for Systematic Enrichment Analysis (IPAD).

Gene	Name
APITD1	ATPase, Na+/K+ transporting, beta 1 polypeptide
ASIC1	acid-sensing (proton-gated) ion channel 1
**AURKA**	aurora kinase A
**CASP9**	caspase 9, apoptosis-related cysteine peptidase
**CCNB1**	cyclin B1
**CD274**	CD247 molecule
**CDK1**	cyclin-dependent kinase 1
CX3CL1	chemokine (C-X3-C motif) ligand 1
FN1	fibronectin 1
**HGF**	hepatocyte growth factor
**ITGA2**	integrin, alpha 2
**KIF20B**	kinesin family member 20B
**LCK**	lymphocyte-specific protein tyrosine kinase
NCF2	neutrophil cytosolic factor 2
PRKCB	protein kinase C, beta
**PSD**	pleckstrin and Sec7 domain containing
**STAT3**	signal transducer and activator of transcription 3 (acute-phase response factor)
**TRAC**	T cell receptor alpha constant
**WARS**	tryptophanyl-tRNA synthetase

Pathway analysis in IPAD generated 18 enriched pathways, 4 of which (cell cycle (mitotic), immune system, hemostasis and metabolism) were shared with those of MeSH ID’s for hypertension and cardiovascular disease (Table 5). While no pathways identified by IPAD were associated with CoA, one individual DEG from the current investigation, ATP2A1, associated with Aortic Coarctation in IPAD was also located in the Hemostasis pathway. A review the available literature for each highly DEG, and their potential involvement in sources of CV morbidity is provided in the discussion section.

**Table 5:** 18 pathways containing ≥5 DEGs with >6FC were found using the Integrated Pathway Analysis Database for Systematic Enrichment Analysis (IPAD). Of these pathways, 5 (bolded) are associated with Hypertension (HTN; MESH:D006973) and Cardiovascular Disease (CVD; MESH:D002318). Italicized DEG for these 5 bolded pathways were enriched for the CoA group, but not the Corrected group. None of these pathways were associated with Aortic Coarctation (MESH:D001017), but one DEG (ATP2A1) associated with Aortic Coarctation was located in the Hemostasis pathway.

PathwayID	Pathway Name	DEG	# DEG	Pvalue	Assoc. w HTN	Assoc. w CVD	Assoc. w CoA	RE	N	MJI
1640170	**Cell Cycle**	**CDC20, CDK1, KIF23, CCNB2, BUB1B, SKA1, NUF2, AURKB, TOP2A, UBE2C, *PRKAR2B*, *GINS1*, *MCM4***	**10**	**0.0002325**	**X**	**X**		7.9	410	0.1511
69278	**Cell Cycle, Mitotic**	**CDC20, CDK1, KIF23, CCNB2, BUB1B, SKA1, NUF2, AURKB, TOP2A, UBE2C, *PRKAR2B*, *GINS1*, *MCM4***	**10**	**0.000149**	**X**	**X**		9.79	331	0.154
168256	**Immune System**	**CDK1, ITGA4, LCK, CD247, CD3G, CDC20, GBP4, UBE2C, TRAC, TRAT1, *MKRN1*, *CD36***	**10**	**0.0077407**	**X**	**X**		3.67	882	0.1446
1280218	Adaptive Immune System	CD247, CD3G, ITGA4, CDC20, LCK, UBE2C, TRAT1, TRAC	8	0.002674873				5.38	482	0.1194
69306	DNA Replication	AURKB, NUF2, SKA1, BUB1B, KIF23, CDC20	6	0.001011944				9.72	200	0.0983
109582	**Hemostasis**	**ITGA4, KIF11, KIF23, LCK, ATP2A1, KIF4A, *PRKAR2B*, *CD36*, *KIF15*, *HRG***	**6**	**0.0260163**	**X**	**X**		4.14	469	0.0897
1430728	**Metabolism**	**UCP1, APOA2, TRAC, AMPD1, SLC27A2, *PDX1*, *CYP2B6*, *FABP1*, *ALDOC*, *DGAT2*, *PCK2*, *MGST1*, *PFKFB1*, *PGD*, *CD36*, *DTYMK*, *ALAS2*, *PPARG*, *PRKAR2B***	**5**	**0.3351984**	**X**	**X**		20.25	96	0.1146
68886	M Phase	KIF23, CDC20, AURKB, BUB1B, NUF2, SKA1	6	0.000152332				10.92	178	0.1002
453277	Mitotic M-M/G1 phases	AURKB, BUB1B, NUF2, SKA1, CDC20, KIF23	6	0.00079528				19.75	82	0.0999
174143	APC/C-mediated degradation of cell cycle proteins	CDC20, CDK1, BUB1B, UBE2C, AURKB	5	0.000368985				13.06	124	0.0896
hsa04110	Cell cycle	CDK1, CCNB2, CDC20, BUB1B, TTK	5	0.001011944				13.84	117	0.0908
69620	Cell Cycle Checkpoints	UBE2C, CDC20, CDK1, BUB1B, CCNB2	5	0.000928061				31.15	52	0.1175
202424	Downstream TCR signaling	LCK, TRAC, TRAT1, CD247, CD3G	5	0.000152332				5.96	272	0.0786
hsa05166	HTLV-I infection	CD3G, BUB1B, CDC20, LCK, CCNB2	5	0.012760355				1.43	1134	0.0716
68877	Mitotic Prometaphase	SKA1, NUF2, BUB1B, AURKB, CDC20	5	0.000446311				17.61	92	0.0966
453276	Regulation of mitotic cell cycle	CDK1, CDC20, BUB1B, AURKB, UBE2C	5	0.000368985				19.75	82	0.0999
162582	Signal Transduction	LCK, P2RY10, DEPDC1B, CDK1, ITGA4	5	0.54606175				1.05	1541	0.0711
202403	TCR signaling	CD3G, CD247, TRAT1, TRAC, LCK	5	0.000249534				23.48	69	0.1057

## Discussion

While current treatments for CoA have improved life expectancy, these individuals still suffer a reduced average lifespan and morbidity resulting from CVD. Clarence Crafoord, M.D. performed the first surgical correction for CoA on October 19, 1944 using resection with end-to-end anastomosis[32], and this approach is still among the most commonly implemented surgical techniques. The morbidity that continues to be observed in corrected CoA patients 70 years later, primarily presenting in the form of hypertension, underscores the need to elucidate mechanisms associated with persistent vascular changes after correction for CoA in an effort to improve scientific knowledge and translate this knowledge to clinical practice. The current work describes the most comprehensive list of differentially expressed genes to date in the upstream aorta that is subjected to increases in arterial BP after surgical induction of CoA, and restoration of normal arterial BP after its correction. DEGs may offer additional insight into potential mechanisms of persistent CV morbidity presenting in these patients.

**Fig 7:**
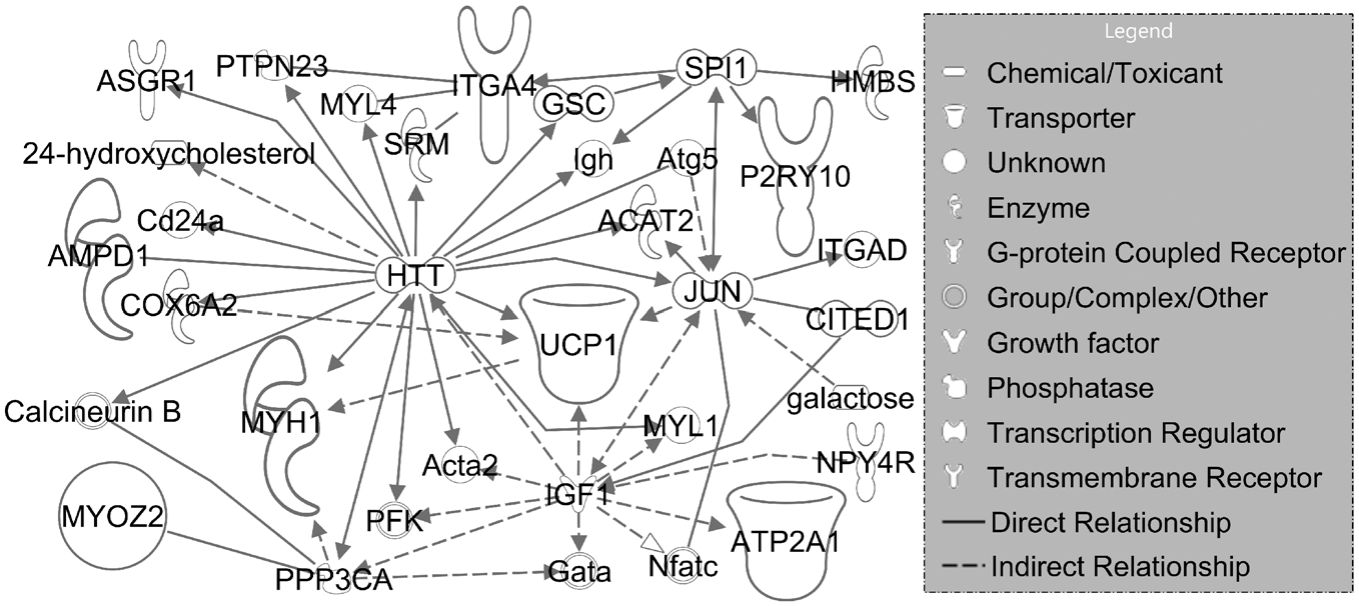
Relationship obtained from IPA for the seven DEGs that may collectively account for the hemodynamic, histological, immunohistochemical and myographic changes observed in our rabbit CoA model to date.

A thorough PubMed search was conducted for each DEG within the enriched pathways that were also associated with diseases commonly presenting in CoA patients (HTN and CVD). The following 7 genes are aligned with the hemodynamic, histological, immunohistochemical and myographic changes observed in our rabbit CoA model to date[12]. A brief summary of some of their known or proposed functions is included below and their relationship is shown in Fig 7.

- *MYOZ2* (also known as Calsarcin-1 and CS-1)—inhibits protein phosphatase 2B activity in the nuclear factor of activated T cells (NFAT) pathway, α-actin and α-filamin binding protein
- *ITGA4*—facilitates activation of L-type voltage-activated Ca^2+^ channels, inside-out and outside-in receptor as well as structural membrane protein involved in force transmission
- *P2RY10*—G-protein coupled purinergic receptors for endothelial-derived nitric oxide (NO) relaxation and other pathways
- *UCP1*—Uncoupling agent regulating ATP in SM contraction and decreasing NO availability, involved in futile cycling to vary heat and ATP production
- *AMPD1*—plays role in muscle metabolism and vasodilation, shifts in energy substrates
- *ATP2A1* (also known as SERCA)—mediates NO function in SM and EC by maintaining intracellular Ca^2+^ levels, regulates membrane Ca^2+^ pump
- *MYH1*—Myosin heavy chain involved in force generation and/or cell shortening

**Fig 8:**
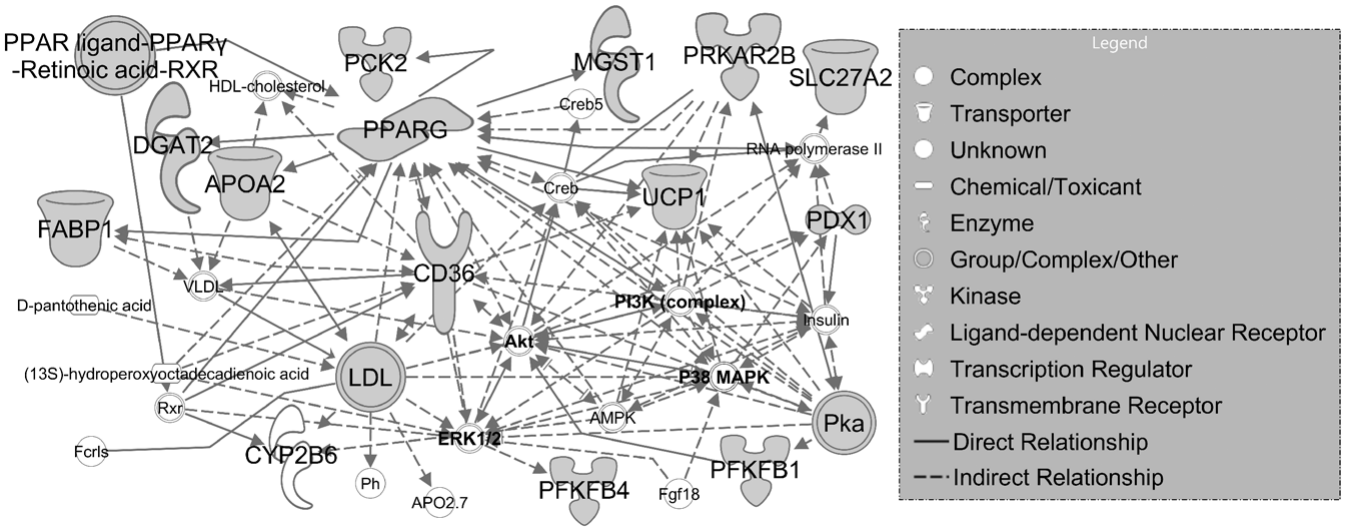
Relationship between DEGs within the metabolism pathway of IPA.

Of these DEGs, AMPD1 and ATP2A2 are particularly interesting in the potential pathogenesis of CoA. Hand et al showed that an adenosine monophosphate deaminase-1 (AMPD1) C34T gene polymorphism detected in blood samples had an impact on vasodilatory response as determined from forearm blood flow measurements[33]. Interestingly no study has determined the presence or severity of AMPD1 C34T mutations in human patients previously undergoing correction for CoA. This may be a particular area of interest given the large number of DEGs within the metabolism pathway of IPAD and IPA, which is shown in Fig 8.

Our results also showed a >6 FC change in ATP2A1, commonly known as sarco/endoplasmic reticulum Ca^2+^ ATPase (i.e. SERCA). SERCA plays a role in mediating NO function in both SMCs and EC[34]. Specifically, SERCA maintains intracellular Ca^2+^ levels in SM within optimal limits by transporting Ca^2+^ into the sarco/endoplasmic reticulum, and NO stimulates SERCA activity. Van Hove and colleagues showed this NO-based stimulation of SERCA occurs by non-cGMP means[35]. A study in hypertensive rats showed regression in transcript levels of SERCA 2a and 2b that were localized to vascular tissue in response to Nifedipine treatment[36]. Collectively these findings suggest administration of Nifedipine, if efficacious, may reduce hypertension in repaired CoA, but this hypothesis remains to be tested and Ca^2+^ channel blockers are known to have a wide variety of effects. In contrast to Nifedipine, there are documented studies of treated CoA patients receiving Enalapril[37] and captopril[38, 39]. Excessive angiotensin II production in streptozotocin-induced diabetic rats can lead to the generation of peroxynitrite, and that this may subsequently trigger dysfunction of vascular SM SERCA, but the adverse impact of these changes is mitigated by Enalapril[40]. Cheng et al., also reported that SERCA is not only required for a cell to exit G_o_ and enter the cell cycle, but that it is also involved in controlling this process[41]. Lompre reported that proliferation of SMCs is associated with changes in intracellular Ca^2+^ handling[42]. Thus there may be a causal relationship between SERCA and failure of SMCs to re-differentiate following correction of the CoA reported in our experimental animals. Bonaventora et al. also reported observing a decrease in NO relaxation in hypertensive rats that is due to a decrease in SERCA activity[43]. This correlation may also be relevant as a possible factor in the decreased EC relaxation we reported proximal to the CoA site in our model system that does not return to normal after correction[12].

A study by Chang et al suggested thyroid hormone (T4) could improve calcium handling by SERCA, as well as alpha-MHC expression, in pressure-overloaded hearts[44]. Studies also indicated that the proportion of L-type Ca^2+^ channels increases during proliferation, in contrast to decreases in T-type, and that these changes are dependent on the cell cycle[42, 45], another pathway with several highly expressed genes in the current investigation. The numerous links to changes in metabolic pathway enzymes (Table 5 and Fig 8), and the many roles of T3 and T4 in cell growth, differentiation, development, and metabolism suggest that there may also be a link between changes in these DEGs and the reported failure of SMCs proximal to the coarctation to re-differentiate, respond to EC-derived relaxation, and generate force in CoA and corrected rabbits[12].

Several additional DEGs of potential interest in the persistent morbidity after correction for CoA were also noted. Integrins are single transmembrane-spanning receptors that are not only receptors for communication, but are also structural proteins that can transmit forces between cells via their connection between the extracellular matrix and the intracellular cytoskeleton[46, 47]. Their close and alternating association with caveoli on the plasma membrane[48], and direct association with cytoskeletal and regulatory proteins, has led to numerous hypotheses suggesting multiple roles in regulating the generation of force in SM[47, 49, 50]. Any changes to integrin expression and or regulation could have significant downstream implications for numerous SM functions, including those reported to change irreversibly in our CoA model[12]. The current results show alpha4 integrin (>6FC down-regulated) and Calsarcin-1 (a.k.a CS-1 and MYOZ2; >8FC up-regulated) are both DEGs in the aortas of CoA and corrected rabbits. There may be translational applications for CoA patients since CS-1 is known to inhibit protein phosphatase 2B activity invoked the calcineurin-NFAT pathway[51], and reports have documented the use of 17β-Estradiol[52] and Ginsenoside Rg_1_[53] as pharmacologic treatment agents targeting this pathway. While both these studies focus on cardiac hypertrophy, studies have also reported the presence of CS-1 in SMCs[54], which is consistent with the current results from rabbit aortas. Similarly, periostin (POSTN), another DEG with >8 FC in the current results, also appears to be a target for reversing the impact of cardiac hypertrophy via the NFAT pathway[55].

CD247 is a gene related to immunity, and the immune system were two of the pathways revealed by IPAD to have the highest number of DEGs with >6 FC (10 and 8 DEGs, respectively). A recent analysis of T-cell trafficking methods and protocols points out that a loss in T-cell receptor zeta, CD247, is common in chronic infectious and inflammatory diseases[56], including an association with systolic BP in single nucleotide polymorphisms in intron 1 of European descendants[57]. More recently, Rudemiller offered data in support of a role for CD247 in hypertension that is mediated by altered immune cell function[58]. The authors surmise that an initial increase in BP as a result of the ingestion of high salt in Dahl salt-sensitive rats leads to a cellular immune response and the infiltration of activated T lymphocytes, which are localized around blood vessels and several other locations, and can participate in the formation of cytokines, free radicals, and angiotensin II. Morgan and colleagues also showed that a mutation in the TCRα subunit constant (TRAC) gene, also down-regulated by >6 FC in our CoA and corrected rabbits, was associated with impaired T cell function leading to infection and autoimmunity[59]. Nonetheless, there is a lack of data on the specific role of C247 or TRAC in persistent morbidity after correction of CoA.

The current results revealed down-regulation of G-protein coupled purinergic receptors (P2Y) within both the CoA vs Control and Corrected vs Control comparisons. P2Y are thought to be necessary for NO relaxation and we previously showed a loss (for CoA rabbits) and reduction (for corrected rabbits) in ACh-induced NO relaxation in for aortic samples within the region experiencing elevated arterial BP.

Uncoupling agents such as UCP1 produce SM contraction[60], a hallmark of HTN. UCP1 expression also increases superoxide production and decreases the availability of NO, resulting in oxidative stress[61]. Here we show that UCP1 expression is down-regulated by >10-fold in corrected rabbits and >20-fold in CoA rabbits. Doxycycline induction of aortic UCP1 expression in rats has been shown to increase tailcuff systolic BP by 46mmHg and diastolic BP by 21mmHg[61]. This further confirms our analysis and may link even a transient increase in hypertensive stimuli from a coarctation to other persistent sources of morbidity in patients treated for CoA.

The current results should be interpreted relative to several potential limitations. The results of microarray analysis in the current model of CoA before and after correction should be considered as hypothesis-generating, as the functional relevance of these candidate genes in human CoA is unknown. In addition, while changes in gene expression can be important, it is protein expression and function that ultimately determines a change in physiology. Changes in gene expression may be compensated for by other mechanisms (e.g. changes in other genes, altered protein degradation rates, etc.). Post-translational modifications of proteins or changes in protein degradation can also occur in the absence of changes in gene expression, and thus may not be detected by microarray analysis. We were unable to reproducibly correlate UCP1 or ITGA4 protein level with respective mRNA expression. A potential lack of a correlation between protein and mRNA expression may also be due to differences in approach. We previously showed changes in matrix and SM markers[12]. In reviewing details of the microarray used here, it appears there are no probes for collagen, but it does have probes for both SM and NM MHC. These probes did not reveal significant changes in expression between groups of control, CoA, and corrected rabbits, which contradicts the decrease in SM MHC and increase in NM MHC protein expression we reported previously using immunohistochemistry[12]. However, on further review we found the MYH9 probe binds to base pairs 1768–1827 of the NM MHC RNA sequence. There is <20% difference between the NM and SM MHC sequence in this region, which could allow for potential binding to the SM sequence. In addition, the MYH11 probe on the microarray binds to base pairs 5956–6015 of the SM MHC message. There are 13 differences between the NM and the SM MHC in this region (with 13 skips). This may be enough difference for the probe to distinguish between the two MHC phenotypes. Perhaps more importantly, this represents the very carboxyl terminal of the MHC protein, and only one of the SM isoforms is actually this long. Thus, the probe would only detect the SM1 carboxyl terminal MHC isoform, but not the SM2 MHC isoform that changes significantly with SMC differentiation/de-differentiation. This could explain why changes in the microarray data for MYH11 were not observed to correlate with the decrease in SM MHC protein expression we observed previously[12].

The current work employs a transcriptomic, rather than proteomic approach. In general, the approach used here is preferred for initial studies where little is known (e.g., CoA), and a potentially long term global change is hypothesized. Ultimately, both approaches will need to be completed to paint a complete picture of how protein expression is regulated and impacts physiological changes in CoA.

The DEGs discussed above represent those resulting from the mechanical stimuli induced by a coarctation. In humans this mechanical stimuli is most likely accompanied by patient-specific causal genetic predispositions that may accentuate, mitigate, or be isolated from those presented here. While human CoA is likely a genetically-created pathology, it is interesting that we can recapitulate the effects of idiopathic CoA in humans by mechanical perturbation in our experimental rabbit model of CoA. An experimentally generated focal constriction of the aorta in our model (or possibly a developmental miscue resulting in an aortic constriction in humans) may be enough to begin a series of positive feedback changes that ultimately result in the localized and systemic changes associated with persistent morbidity often accompanying CoA. Interestingly Pfaltzgraff and colleagues recently showed that vascular SMCs from different embryonic origins manifested in their location within the aorta are functionally distinct in the embryonic mouse, but develop to a common phenotype by adulthood[62]. It seems possible then that the presence of adverse mechanical stimuli, including even a transient increase in BP for a period of weeks that is the focus of the current work, may influence the ultimate fate of vascular SMCs from these distinct origins before they have a chance to terminally differentiate into their adult phenotype.

## Conclusion

The current results of differentially-expressed human orthologue genes from a rabbit model of CoA with novel correction by dissolvable suture revealed many genes previously known to be associated with HTN and CoA in humans[63–67] or animals[68, 69], while also elucidating many others that have not been described or associated with the pathology of CoA (untreated or corrected) to date. Future work will further scrutinize these gene candidates and evaluate accompanying methods aimed at assessing therapeutic or translational potential. Having this experimental model to study CoA, and a list of candidate DEGs, in conjunction with clinical studies should facilitate progress towards alleviating persistent CV morbidity after treatment of CoA.
